# Efficacy and safety of Chinese medicine for obstructive sleep apnea: A protocol for systematic review and meta-analysis

**DOI:** 10.1097/MD.0000000000023903

**Published:** 2021-01-22

**Authors:** Jun-Li Bao, Xinyuan-Gao, Yu-Bo Han, Ke Zhang, Li Liu

**Affiliations:** a Heilongjiang University of Chinese Medicine, No.24, Xiangfang District; b First Affiliated Hospital, Heilongjiang University of Chinese Medicine, Haerbin, Heilongjiang, China.

**Keywords:** Chinese medicine, obstructive sleep apnea, review, meta-analysis

## Abstract

**Background:**

Obstructive sleep apnea (OSA) is significant public concern. Clinical practice indicates that Chinese medicine has certain therapeutic advantages, while there is a lack of evidence-based medicine support. The aim of this study is to synthesize related data to explore efficacy and safety of Chinese medicine for OSA.

**Methods:**

Data in PubMed, Embase, Web of Science, CNKI, WanFang, VIP databases were comprehensively searched. All the randomized controlled trials (RCTs) in OSA children were identified, in which the effects of Chinese medicine on a range of outcomes were compared. The search had a deadline of January 1, 2020. Two investigators independently conducted data extraction and assessed the literature quality of the included studies. The Revman5.3 software was used for meta-analysis of the included literature.

**Results:**

The efficacy and safety of Chinese medicine for OSA were evaluated in terms of apnea hypopnea index (AHI, the average and lowest blood oxygen, the Epworth Sleep Scale [ESS], and adverse effects).

**Conclusions:**

This study provides reliable evidence-based support for the clinical application of Chinese medicine for OSA.

**PROSPERO registration number:**

CRD42020154864.

## 1 Introduction

Obstructive sleep apnea (OSA) mainly features repetitive episodes of cessation (apnea) or reduction (hypopnea) in airflow during sleep caused by upper airway obstruction.^[1]^ About 22% of men and 17% of women have been diagnosed as OSA with apnea hypopnea index (AHI) ≥5/h according to the epidemiological data reported from 1993 to 2013.^[2]^ OSA as a systemic disorder can result in multiple problems, encompassing hypertension, cardiovascular disease, insulin resistance, the increased morbidity and mortality of cancers, and neurodegeneration.^[3]^ Continuous airway positive pressure (CPAP) therapy is the most common treatment for OSA.^[4]^ However, its poor compliance restricts its clinical use and effectiveness.^[5,6]^ These has instigated exploration of non-CPAP therapeutic alternatives.

In recent years, Chinese medicine has been widely used in clinical and experimental studies of OSA.^[7]^ It has been shown to have some advantages and less side effects.^[8]^ Chinese medicine has been used to improve symptoms in OSA patients,^[9]^ but its effectiveness and safety have not yet reached a definitive conclusion. Therefore, this study aimed to systematically assess the efficacy and safety of Chinese medicine for OSA and provide a reliable reference for the clinical application of Chinese medicine for OSA.

## 2 Methods

### 2.1 Protocol register

This protocol of systematic review and meta-analysis has been drafted under the guidance of the preferred reporting items for systematic reviews and meta-analysis protocols (PRISMA-P).^[10]^ Moreover, it has been registered on the PROSPERO on October 26, 2020 (registration number: CRD42020154864).

### 2.2 Ethics

Since this is a protocol with no patient recruitment and personal information collection, approval by the ethics committee is not required.

### 2.3 Eligibility criteria

#### 2.3.1 Types of studies

We will collect all available randomized controlled trials (RCTs) on Chinese medicine for OSA, regardless of publication status, and region, but language will be restricted to Chinese and English.

#### 2.3.2 Patients

Participants included in our study had to be diagnosed with OSA according to the results of polysomnography (PSG) (AHI > 5). No restrictions on age, sex, and race were imposed.

#### 2.3.3 Intervention

Patients in the treatment group received Chinese medicine, while patients in the control group received placebo or no treatment or nCPAP or pharmacological compounds (e.g., such as theophylline) or non-pharmacological interventions (e.g., diet, exercise). There were no restrictions on the dose, frequency, and treatment course of Chinese medicine.

#### 2.3.4 Outcome indicators

(1) Main outcomes: The primary modes of measurement were the PSG monitoring indicators and the Epworth Sleep Scale (ESS). The monitoring indicators of PSG include AHI index,^[11]^ the average and lowest blood oxygen.^[12]^ The ESS is composed of 8 situational questions used to evaluate the severity of daytime sleepiness.^[13]^ Each question was worth 0 to 3 points, so the total ESS scores that each patient earned ranged from 0 (best) to 24 (worst).
(2) Additional outcomes: Adverse effects (numbers of participants experiencing each adverse events).

### 2.4 Exclusion criteria

(1) Animal experiments, reviews, case reports, and repetitive publications or search outcomes.
(2) The published papers were abstracts or the data were incomplete and the papers with complete data were not available after contacting the author.
(3) Papers containing <10 cases.
(4) Papers assessed as high risk of bias by randomization or concealed distribution.^[14]^
(5) Papers with no relevant outcome indicators.

### 2.5 Information sources

The search will use a sensitive subject and topic-based strategy from inception to January 1, 2020. Databases searched will be PubMed, Embase, Web of Science, China National Knowledge Infrastructure (CNKI), Wan-Fang Database, and China Science and Technology Journal Database (VIP).

### 2.6 Search strategy

Take “Chinese medicine (zhong yao),” “traditional chinese medicine (zhong yi yao),” “obstructive sleep apnea (zu sai xing shui mian hu xi zan ting),” “snoring (da han)” as the Chinese search terms and search in Chinese databases, including CNKI, Wanfang, VIP. Take “Chinese medicine,” “traditional chinese medicine,” “obstructive Sleep Apnea,” “obstructive sleep apnea syndrome,” “obstructive sleep apneas” as the English terms and search terms and search in English databases, including PubMed, EMBASE, Web of Science were searched manually. Taking PubMed as an example, the retrieval strategy is shown in Table 1.

**Table 1:** Search strategy for the PubMed database.

#1	Chinese medicine
#2	Chinese drug
#3	traditional chinese medicine
#4	chinese traditional medicine
#5	#1 OR #2 OR #3 OR #4
#6	obstructive sleep apnea
#7	sleep disordered breathing
#8	obstructive sleep apnea syndrome
#9	Upper Airway Resistance Sleep Apnea Syndrome
#10	obstructive sleep apnea
#11	OSAHS
#12	Syndrome, Upper Airway Resistance, Sleep Apnea
#13	Syndrome, Sleep Apnea, Obstructive
#14	Sleep Apnea Syndrome, Obstructive
#15	Apnea, Obstructive Sleep
#16	Sleep Apnea Hypopnea Syndrome
#17	Syndrome, Obstructive Sleep Apnea
#18	#6 OR #7 OR #8 OR #9 OR #10 OR #11 OR #12 OR #13 OR #14 OR #15 OR #16 OR #17
#19	#5 AND #18

### 2.7 Data filtering and extraction

**Figure 1:**
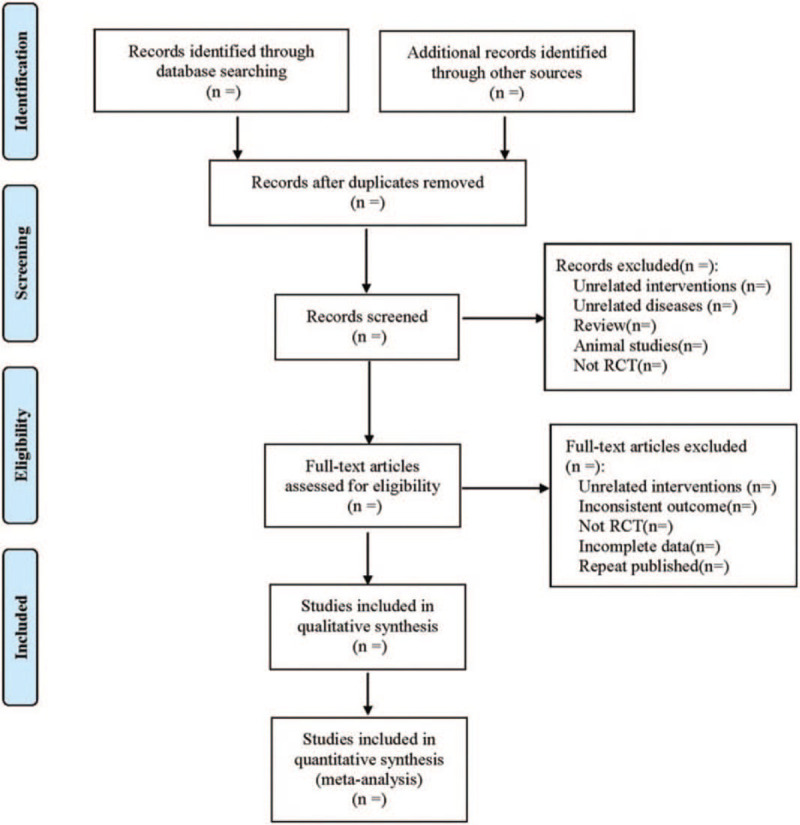
The process of literature filtering.

Referring to the method of research selection in version 5.0 of the Cochrane Collaboration Network System Evaluator Manual, according to the preferred reporting items for systematic reviews and meta-analysis (PRISMA) flow chart, 2 authors used the EndNote V.X9 document management software to independently screen and check the literature according to the above inclusion and exclusion criteria, and check each other. A third reviewer reviewed the extracted data and stored the original data in a secure computer to avoid additional changes. At the same time, Excel 2016 was used to extract relevant information, including: the variables, namely, article title, author(s), journal title, year of publication, sample size, sex, age, interventions, the duration of treatment, and outcome. The process of literature filtering is shown in Fig. 1.

### 2.8 Literature quality(bias) assessment

Two reviewers will independently assess the methodological quality of the included RCTs using the Cochrane Collaboration's risk of bias tool,^[15]^ which consists of 7 items: random sequence generation; allocation concealment; blinding of participants and personnel; blinding of outcome assessment; incomplete outcome data; selective reporting, and other sources of bias. Each item is judged as being either “high risk,” “low risk,” or “unclear” risk of bias. “Low risk of bias” means that it is unlikely that the study includes a bias which would seriously alter the results; “high risk of bias” means that it is plausible that a bias would seriously weaken confidence in the results; and “unclear risk of bias” means that it is plausible that a bias raises some doubt about the results. Discrepancies in the interpretation will be resolved by consensus, or by consultation with a third party.

### 2.9 Statistical analysis

#### 2.9.1 Data analysis and processing

Studies that met the inclusion criteria and were similar in intervention were pooled to conclude a meta-analysis. For continuous data, mean differences between the groups and Standard Deviations of the change were used and presented as random effects 95% confidence intervals (CIs) in forest plots. Cochran chi-squared test and the *I*^2^ statistic were applied to estimate the percentage of variability across studies due to between-study heterogeneity. Heterogeneity was considered statistically significant at *P* < .10 and *I*^2^ > 50%. The weighted mean difference (WMD) with 95% confidence intervals (CIs) was calculated using a fixed-effects model if *I*^2^ < 50% and using a random-effects model if *I*^2^ > 50%. Fisher z test was used to determine the statistical significance of the pooled WMDs.

#### 2.9.2 Analysis of subgroups

To investigate specific factors affecting the overall efficacies of the included RCTs, we will also perform subgroup analyses on treatment duration, sample size, publication year, diagnostic criteria, publication language, and Traditional Chinese Medicine (TCM) syndrome.

#### 2.9.3 Dealing with missing data

If there are missing data in the article, contact the author via email for additional information. If the author cannot be contacted, or the author has lost relevant data, descriptive analysis will be conducted instead of meta-analysis.

#### 2.9.4 Sensitivity analysis

In order to test the stability of meta-analysis results of indicators, a one-by-one elimination method will be adopted for sensitivity analysis.

#### 2.9.5 Assessment of reporting biases

Publication bias was assessed by funnel plot and Egger test. Egger test was performed for no <10 studies, whereas the trim-and-fill analysis was conducted in the presence of publication bias.

#### 2.9.6 Evidence quality evaluation

The Cochrane Collaboration's risk of bias tool will be used to assess the quality of evidence. The quality of evidence will be rated as high, moderate, low, and very low.

## 3 Discussion

Treatments for OSA incorporate CPAP therapy, surgical therapy, and implantable and wearable devices.^[16]^ CPAP therapy is considered as the gold-standard treatment of OSA. Though CPAP achieves favorable therapeutic efficacy, the low patient compliance ranging from 29% to 81%, poses a challenge to medical researchers.^[17]^ Non-CPAP therapies (e.g., oral appliance therapy and upper airway surgery) also achieve positive results in many cases, but their therapeutic efficacy is unstable.^[18,19]^ Mandibular advancement devices even have negative impacts on the oral structure, resulting in changes in the strength and speed of a bite.^[20]^ Thus, new approaches to treat OSA are required.

A growing body of research have shown that Chinese medicine can improve the clinical efficacy of OSA patients. This study aimed to systematically assess the efficacy and safety of Chinese medicine for OSA and provide a reliable reference for the clinical application of Chinese medicine for OSA. However, this systematic review had some limitations: there were differences in the doses of Chinese medicine used in the included studies and the condition of the patient's disease. There may be some clinical heterogeneity. The course of disease was also different, which may have affected the results to some extent. Constrained by language ability, we only searched English and Chinese literature and may ignore studies or reports in other languages.

## Author contributions

**Conceptualization:** Junli Bao.

**Data collection:** Xinyuan Gao.

**Data curation:** Xinyuan Gao.

**Funding acquisition:** Li Liu.

**Investigation:** Yubo Han.

**Literature retrieval:** Ke Zhang.

**Software operating:** Junli Bao.

**Software:** Xinyuan Gao, Junli Bao.

**Supervision:** Yubo Han.

**Writing – original draft:** Junli Bao and Xinyuan Gao.

**Writing – review & editing:** Junli Bao and Li Liu.
